# Hantaan Virus Infection Induces CXCL10 Expression through TLR3, RIG-I, and MDA-5 Pathways Correlated with the Disease Severity

**DOI:** 10.1155/2014/697837

**Published:** 2014-02-23

**Authors:** Yusi Zhang, Bei Liu, Ying Ma, Jing Yi, Chunmei Zhang, Yun Zhang, Zhuwei Xu, Jiuping Wang, Kun Yang, Angang Yang, Ran Zhuang, Boquan Jin

**Affiliations:** ^1^Department of Immunology, The Fourth Military Medical University, 169 Changle West Road, Xi'an 710032, China; ^2^Department of Blood Transfusion, Xijing Hospital, The Fourth Military Medical University, Xi'an 710032, China; ^3^Department of Infectious Disease, Tangdu Hospital, The Fourth Military Medical University, Xi'an 710032, China

## Abstract

Hantaan virus (HTNV) is a major agent causing hemorrhagic fever with renal syndrome (HFRS). Although the pathogenesis of HFRS is unclear, some reports have suggested that the abundant production of proinflammatory cytokines and uncontrolled inflammatory responses may contribute to the development of HFRS. CXCL10 is one of these cytokines and is found to be involved in the pathogenesis of many virus infectious diseases. However, the role of CXCL10 in the pathogenesis of HFRS and the molecular regulation mechanism of CXCL10 in HTNV infection remain unknown. In this study, we report that CXCL10 expresses highly in the HFRS patients' sera and the elevated CXCL10 is positively correlated with the severity of HFRS. We find that HTNV, a single-strand RNA virus, can act as a double-strand RNA to activate the TLR3, RIG-I, and MDA-5 signaling pathways. Through the downstream transcription factors of these pathways, NF-**κ**B and IRF7, which bind directly to the CXCL10's promoter, the expression of CXCL10 is increased. Our results may help to better understand the role of CXCL10 in the development of HFRS and may provide some novel insights into the immune response of HTNV infection.

## 1. Introduction

Hantaan virus (HTNV) is a member of the enveloped *Bunyaviridae* family characterized by a tripartite single-stranded RNA genome of negative polarity. The large (L) segment encodes the viral RNA-dependent RNA polymerase (RdRp), the medium (M) segment encodes two surface glycoproteins (Gn and Gc), and the small (S) segment encodes the nucleocapsid protein (NP) [1]. HTNV could cause a severe lethal hemorrhagic fever with renal syndrome (HFRS) in human [2, 3].

At present, the pathogenesis of HFRS is still unclear. Previous reports suggested that “cytokine storm,” additional immune responses, complement activation, and platelet dysfunction may be the potential mechanisms of HFRS pathogenesis [4].

Inflammatory cytokines/chemokines produced during HTNV infection represent a double-edged sword [5]. On one hand, they contribute to viral elimination by inducing and amplifying innate effectors' functions and antigen presentation of viral epitopes to T cells. On the other hand, if not properly regulated, inflammatory cytokines/chemokines may facilitate immunopathological processes in HFRS [5]. We screened a panel of cytokines expressions using Luminex and found that, in the serum from HFRS patients, CXCL10 was the most-highly-expressed cytokine (see Table S1 in the Supplementary Material available online at http://dx.doi.org/10.1155/2014/697837). CXCL10/Interferon-*γ*-inducible protein-10 (IP-10) is a member of CXC chemokines, which is mainly secreted by endothelial cells, epithelial cells, and keratinocytes. After binding to its receptor CXCR3, CXCL10 induces chemotaxis, apoptosis, cell growth inhibition, and angiostasis [6]. Accumulating data have indicated that CXCL10 plays an important role in many virus infectious diseases infected with Hepatitis Virus B (HBV) [7], Hepatitis Virus C (HCV) [8], HIV [9], influenza virus [10], and some other viruses [11–13]. The elevated level of CXCL10 has been found to be correlated with the development and the severity of these infectious diseases. In studies of the regulation mechanism of CXCL10 production, some signaling pathways have been documented to contribute to the regulation of CXCL10's expression, including JAK-STAT pathway [14], TRAF2/TAK1 pathway [7], and PI3K/AKT pathway [15]. Transcriptional factors like NF-*κ*B [7], IRF1 [13], and IRF3 [15, 16] have also been proved to bind to the CXCL10's promoter. Although Geimonen et al. have reported that HTNV induce CXCL10 expression in HUVECs [17], little is known about the molecular regulation mechanism of HTNV-induced CXCL10 production. A better understanding of the regulation mechanism of CXCL10 production in infectious diseases might help us develop new therapeutic interventions in human diseases.

In this study, we quantified the serum CXCL10 levels in HFRS patients of different severities and in different disease stages, analyzed the relationship between CXCL10 and the disease severity-indicating parameters in vivo, and explored the underlying regulation mechanisms of CXCL10's expression in vitro during HTNV infection.

## 2. Materials and Methods

### 2.1. Ethics Statement

The study was approved by the Institutional Review Board of the Fourth Military Medical University. Written informed consent was obtained directly from each adult subject, and all the children had informed consent given from their guardians for the collection of samples and subsequent analysis.

### 2.2. Study Subjects and Sample Collection

Enrolled in this study were 121 hospitalized patients with HTNV infection from Tangdu Hospital of the Fourth Military Medical University (Xi'an, China) between 2009 and 2011. The clinical diagnosis of HFRS was confirmed by detection of IgM antibodies to HTNV in the patients' serum specimens. Thirty-six healthy donors were included in the study as normal control. The serum samples and the peripheral blood samples were collected and stored as previously described [18, 19]. Based on the classically defined 5 stages of HFRS (namely, febrile stage, hypotensive stage, oliguric stage, polyuric stage, and convalescent stage), we classified the HFRS patients in this study into acute phase (including febrile, hypotensive, and oliguric stages) and convalescent phase (including diuretic and convalescent stages) [20, 21]. The plasma viral load, an important indicator of disease severity, had been determined in our lab before [18]. Because of the limitation in detecting the viral load, we grouped viral load at the baseline of 1500 copies/mL.

### 2.3. Cells and Virus

Human umbilical vein endothelial cells (HUVECs) were prepared by the method described before [22]. Cultures of HUVECs were grown on fibronectin-coated plates (Millipore, USA), were maintained in EGM (Lonza, USA) supplemented with 10% fetal bovine serum (PAA, Austria), 100 IU of penicillin/mL, and 100 *μ*g of streptomycin/mL, and were used before the 10th passage.

HTNV strain 76–118 was propagated on Vero E6 cells. Supernatant was collected from cell cultures at 14 days postinfection and was cleared of cell debris by centrifugation at 2,000 ×g and then filtration by 0.45 *μ*m filter. The stocks of HTNV were aliquot and frozen at −80°C. Mock HTNV control was prepared by subjecting HTNV to Co 60 radiation (1 × 10^4^ Gy). For all infections, virus was allowed to adsorb to HUVECs at multiplicity of infection (MOI) of approximately 1 in serum-free EGM maintenance medium for 2 h at 37°C. The cells were then washed and afterwards incubated in EGM growth medium with 10% FBS. The proportion of infected HUVECs was tested by using immunofluorescence. After 48 h postinfection, over 90% of HUVECs expressed viral nucleocapsid protein in the cytoplasm.

### 2.4. RNA Extraction and Real-Time PCR

The total RNA of HUVECs was extracted using TRIzol (Invitrogen) according to the manufacturer's protocol and 1 *μ*g was used for cDNA synthesis (Takara, Japan). Quantitative analysis of mRNA expression was done by quantitative Real-time PCR using the SYBR Green detection method. The specific primers for each gene were shown in the supporting information Table S2. Reactions were analyzed using a BIO-RAD system (CFX96 Real-Time System). The delta ct method was used to calculate each gene of interest. Then, each gene was normalized to the housekeeping gene GAPDH and was presented as copies of normal medium control for HUVEC studies.

### 2.5. Dual Luciferase Assays

A series of CXCL10 promoter constructs were prepared with the primers shown in the supporting information Table S3 following the previous reports [7]. Point mutations in two NF-*κ*B sites, NF-*κ*B1 and NF-*κ*B2, were generated in the −267/+97 construct as indicated before [7]. The DNA sequencing results showed that all the plasmids were constructed successfully.

HUVECs were plated in a 48-well plate. When the confluence was 70%, cells were transfected with 0.4 *μ*g of each CXCL10 promoter reporter constructs and 5 ng of the pRL-TK plasmids using Lipofectamine (lipo) 2000 (Invitrogen, USA) as manufacturer's protocol. Forty-eight hours later, luciferase activity in each sample was detected with the Dual-luciferase reporter assay system (Promega, USA) according to the manufacturer's instructions and the transfection efficiency was normalized by Renilla luciferase activity. For data analyses, pGL3-basic vector control was set as 1.

### 2.6. RNA Interference

For specific knock down of TLR3, RIG-I, MDA-5, p65, and IRF7, a series of double-strands small interfering RNA were synthesized in Gene Pharma (Shanghai, China) with the sequences as shown in supporting information (Table S4). The transfection of the siRNA into HUVECs was performed using lipo 2000 following the manufacturer's protocol. The validity and efficiency of the siRNA was identified by determining the mRNA and protein levels of corresponding target genes after transfecting the siRNA or the mock vector into HUVECs.

### 2.7. ELISA

The amounts of CXCL10 present in HFRS patients' sera and culture supernatants were determined using a specific mouse anti-human CXCL10 ELISA kit (BD Biosciences, USA) according to the manufacturer's instructions.

### 2.8. Western Blot (WB) Analysis

Total proteins, nuclear proteins and cytoplasmic proteins were prepared as previously described [23], separated by stacking gel and 10%–12% SDS-PAGE separating gel with Tris-glycine system, and transferred onto nitrocellulose membranes (Millipore, USA). The membranes were blocked in 5% skimmed milk and then probed overnight at 4°C with antibodies specific, respectively, to FLAG tag from Sigma, USA; TLR3, MDA-5, and IRF7 from Epitomics, USA; RIG-I, p65, p-I*κ*B, p-Ikk, p-IRF7, *β*-tubulin, and Histone H3 from Cell Signaling Technology, USA; and GAPDH from Ambion, USA. The membranes were washed and incubated with HRP-conjugated goat anti-mouse antibody or HRP-conjugated goat anti-rabbit antibody (Pierce, USA). After washing the membranes, the blots were developed using electrochemiluminescence (Alpha Innotech, USA).

### 2.9. Electrophoretic Mobility Shift Assay (EMSA)

Nuclear extracts were prepared as previously described from HUVECs infected with HTNV for 24 h and 48 h, respectively. Biotin-labeled oligonucleotides were synthesized with the following sequences, for NF-*κ*B1 site, (5′-CATGGGACTTCCCCAGGAACAGC-3′ and 5′-GCTGTTCCTGGGGAAGTCCCATG-3′) and for ISRE site (5′-ATGTTTTGGAAAGTGAAACCTAATTC-3′ and 5′-GAATTAGGTTTCACTTTCCAAAACAT-3′). The super shift assay was performed by the addition of anti-p50 antibody (cell signaling technology, USA), anti-p65 antibody, and anti-IRF7 antibody to the binding reaction. The specificity of protein-DNA interaction was confirmed by unlabeled competition probes of the same sequence or the NF-*κ*B1 mutated probes (5′-CATGtGACTTCaCCAGGAACA-3′) and IRF7 mutated probes (5′-ATGTTTTGGAAgGTGAAgCCTAATTCA-3′).

### 2.10. Chromatin Immunoprecipitation (ChIP) Assay

ChIP assay was conducted using a commercial ChIP assay kit (Upstate Biotechnology, USA). Briefly, HUVECs infected with HTNV for 48 h were cross-linked using 1% formaldehyde at RT for 10 min. After being washed by cold PBS, cells were scraped into a conical tube and sonicated to shear the chromatins. The sonicated chromatins were incubated with anti-p50 antibody, anti-p65 antibody, or anti-IRF7 antibody overnight at 4°C with gently shaking. The normal mouse IgG was set as an isotype control and the anti-RNA polymerase was set as a positive control. Then, the DNA-chromatin-antibody complexes were collected with protein G agarose beads. Afterwards, the DNA was purified and subjected to PCR amplification using the following primers flanking the NF-*κ*B1 site (5′-AACTTGGAGGCTACAATAAA-3′and 5′-GAGGAATGTCTCAGAAAACG-3′) and ISRE site (5′-TCTATATGCAATGAAGTTCT-3′ and 5′-AAGGGCATTACAGTTGACTT-3′) in *CXCL10* promoter, respectively.

### 2.11. Flow Cytometry (FCM) Assay

PBMCs of HFRS were isolated and resuspended at a concentration of 10^7^cells/mL in flow buffer (PBS + 1% FCS + 0.1% NaN3). Fc receptors were blocked by the addition of normal goat serum. Then, 10^6^ cells were stained for 30 minutes at 4°C with monoclonal antibodies (allophycocyanin (APC) conjugated anti-CXCR3 and fluorescein isothiocyanate (FITC) conjugated anti-CD14 purchased from BD Biosciences) and with isotype controls (APC-conjugated and FITC-conjugated mouse IgG1 purchased from eBioscience). Cells were washed twice with flow buffer and 200 *μ*L of 4% formalin was added to fix the cells. A minimum of 100,000 cells were acquired on a BD FACS Calibur Flow Cytometer (Beckman Coulter, Fullerton, CA) and analyzed using Flowjo software (Treestar, Ashland, OR).

### 2.12. Data Analysis

The analysis was performed by SPSS and GraphPad Prism5 software. The statistical significance was determined using One-way ANOVA. The Spearman correlation test was used to test the correlation between CXCL10 concentrations and clinical parameters. A *P* value less than 0.05 was considered to be statistically significant.

## 3. Results

### 3.1. Elevated CXCL10 Level in HFRS Patients' Sera Is Positively Correlated with HFRS Severity

**Figure 1:**
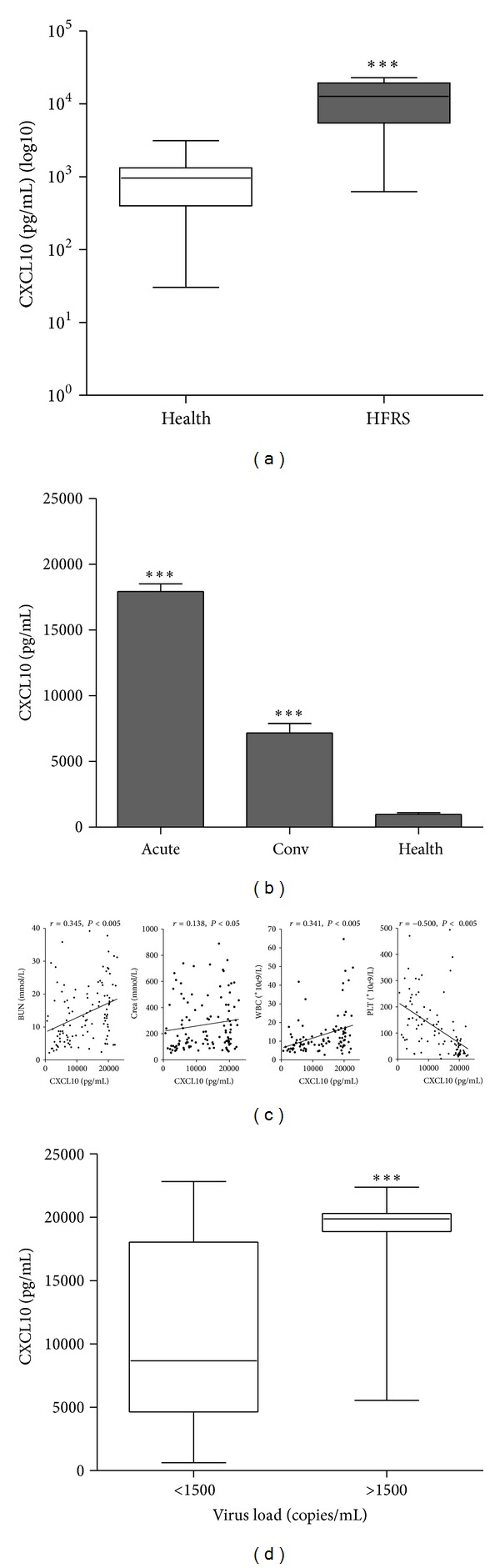
*Increased CXCL10 level in HFRS patients' sera is positively correlated with HFRS severity.* (a) Box plot displaying the minimum, 25th, 50th, 75th, and maximum percentiles of CXCL10 levels. Comparison of serum CXCL10 contents between healthy donors (NC) and HFRS patients. Data were means ± SE (NC, *n* = 36; HFRS, *n* = 121). ***P < 0.001, HFRS patients versus healthy donors. (b) Contents of CXCL10 in acute and convalescent phases in HFRS patients. Data were means ± SE (NC, *n* = 36; HFRS, *n* = 121). ***P < 0.001, acute or convalescent phase versus NC. (c) Serum CXCL10 levels were positively correlated with blood urea nitrogen (BUN), creatinine (Crea), and white blood cells (WBC) in the entire group of subjects while negatively correlated with platelet count (PLT). The *r* and *P* values were indicated in the graphs. (d) Serum CXCL10 levels were in relation to the plasma viral loads. Patients with viral loads >1500 copies/mL had significant higher CXCL10 levels than those with viral loads <1500 copies/mL (***P < 0.001).

The mean level of CXCL10 in HFRS patients was 12.35 times higher than that in normal control (12000.62 ± 632.78 versus 971.49 ± 123.42, *P* < 0.001) (Figure 1(a)). The CXCL10 content in the acute phase was higher than that in the convalescent phase in HFRS patients (*P* < 0.001) and the CXCL10 levels in both acute phase and convalescent phase in HFRS patients were significantly higher compared with those in normal control (*P* < 0.001) (Figure 1(b)). Spearman correlation analysis showed that the increasing level of CXCL10 was correlated with the increasing BUN (*r* = 0.345, *P* < 0.005), Crea (*r* = 0.138, *P* < 0.05), and WBC (*r* = 0.341, *P* < 0.005) and the decreasing PLT counts (*r* = −0.500, *P* < 0.005) in HFRS patients (Figure 1(c)). The CXCL10 levels in viral loads >1500 copies/mL group were significantly higher than those in viral loads <1500 copies/mL group (*P* < 0.001) (Figure 1(d)).

### 3.2. CXCR3 Expression Is the Highest in Acute Phase of HFRS

**Figure 2:**
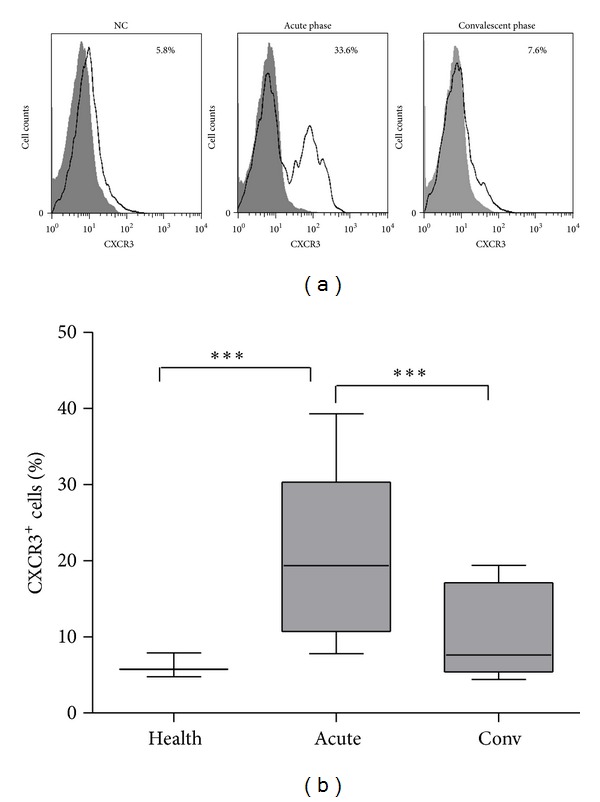
The expression level of *CXCR3* increases highly in acute phase of *HFRS*. (a) The levels of CXCR3 expression on CD14^+^ subsets in PBMCs were measured by flow cytometry. The CD14^+^ cells were gated. The expressions of CXCR3 on CD14 population were presented from a healthy donor (NC), a HFRS patient suffering firstly from acute phase, and then from the convalescent phase, respectively. Shaded histograms represented the isotype controls; black lines represented the expression of CXCR3 on CD14 gated PBMCs. The percentage of CXCR3^+^ cells in the CD14^+^ subsets was indicated in each graph. (b) The percentages of CD14^+^CXCR3^+^ cells in all the CD14^+^ subsets in PBMCs were shown in different groups. Values that are statistically different (*P* < 0.001) between NC and acute phase or between acute phase and convalescent phase are indicated (***).

Flow cytometry analysis showed that the expression of CXCR3, *the receptor of CXCL10*, on CD14^+^ subset was increased in the acute phase of HFRS (Figure 2(a)). The percentage of CD14^+^CXCR3^+^ cells in all the CD14^+^ subset in PBMCs increased from (6.13 ± 0.92) % (normal control, *n* = 4) to (21.00 ± 6.21) % (HFRS patients in acute phase, *n* = 16) and went back to (10.17 ± 2.28) % (HFRS patients in convalescent phase, *n* = 9) (Figure 2(b)). Values were statistically different between normal control and HFRS patients in acute phase and between HFRS patients in acute phase and patients in convalescent phase (both, *P* < 0.001).

### 3.3. HTNV Infection Activates TLR3, RIG-I, and MDA-5 Pathways

**Figure 3:**
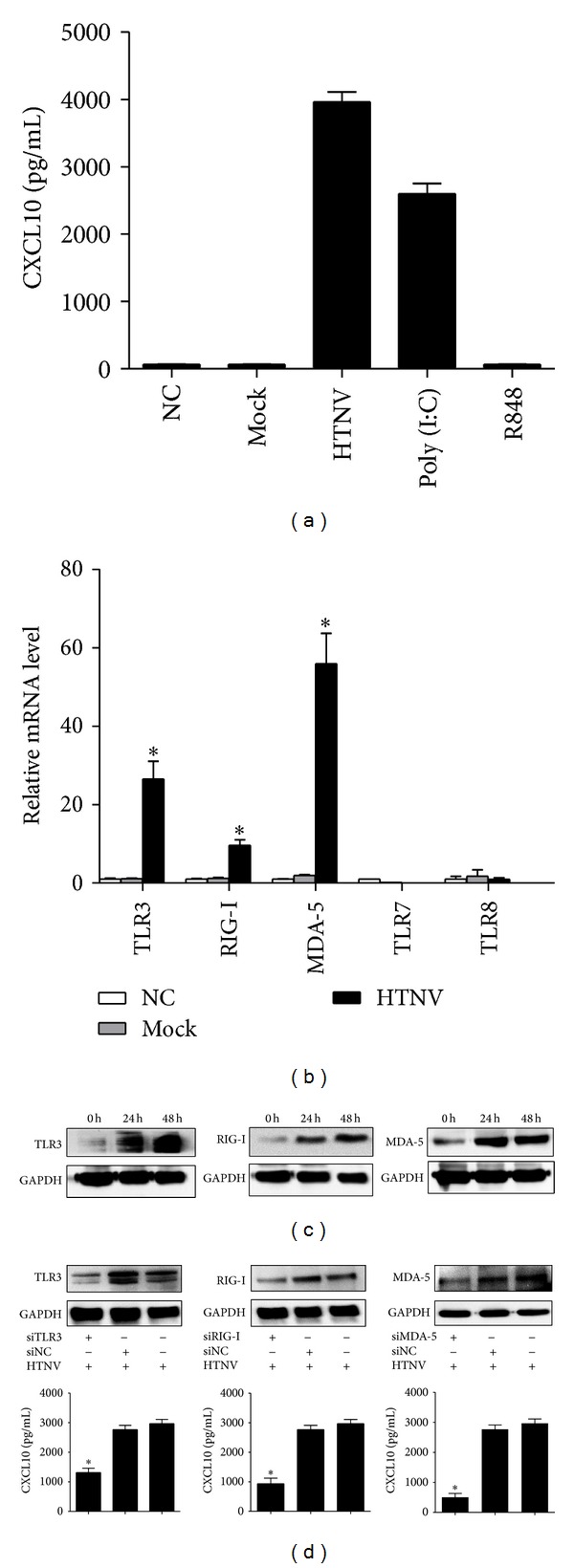
*HTNV infection activates TLR3, RIG-I, and MDA-5 signaling pathways.* (a) In HUVEC model, both the HTNV infection and poly (I:C) stimulation for 48 h can induce the expression of CXCL10. However, mock HTNV infection and R848 treatment for 48 h failed to raise the expression of CXCL10. (b) Real-time PCR screened out that TLR3, RIG-I, and MDA-5 upregulated after HTNV infection for 48 h. (c) WB results indicated that the protein expressions of TLR3, RIG-I, and MDA-5 increased at 24 h and 48 h post-HTNV infection. (d) When transfected into HUVECs for 24 h with siRNA (upper panel), specific to TLR3, RIG-I, or MDA-5, respectively, and then infected the cells with HTNV (MOI = 1) for another 48 h, the expressions of CXCL10 reduced (lower panel). All the experiments were repeated more than three times. mRNA data were generated from three independent experiments with three independent HUVECs donors. Data were means ± SE. *P < 0.05, HTNV infected or poly (I:C) treated versus medium treated cells; and siRNA specific to TLR3, RIG-I, or MDA-5, versus siNC treated cells.

ELISA detection demonstrated that the secretion of CXCL10 was most robust after the HUVECs was transfected with 5 *μ*g/mL poly (I:C) or was infected by HTNV (Figure 3(a)). Real-time PCR results suggested that TLR3, RIG-I, and MDA-5, the sensors of viral dsRNA, were upregulated after HTNV infection, while TLR7 and TLR8, the sensors of ssRNA, remained unchanged (Figure 3(b)). The protein levels of TLR3, RIG-I, and MDA-5 were all elevated with the extension of the infection duration (Figure 3(c)). Transfection of siTLR3, siRIG-I, and siMDA-5, respectively, into HUVECs demonstrated that the knocking down of these dsRNA sensors also cut off the expression levels of CXCL10 induced by HTNV infection (Figure 3(d)).

### 3.4. NF-*κ*B1 and IRF7 Upregulate CXCL10 Expression

**Figure 4:**
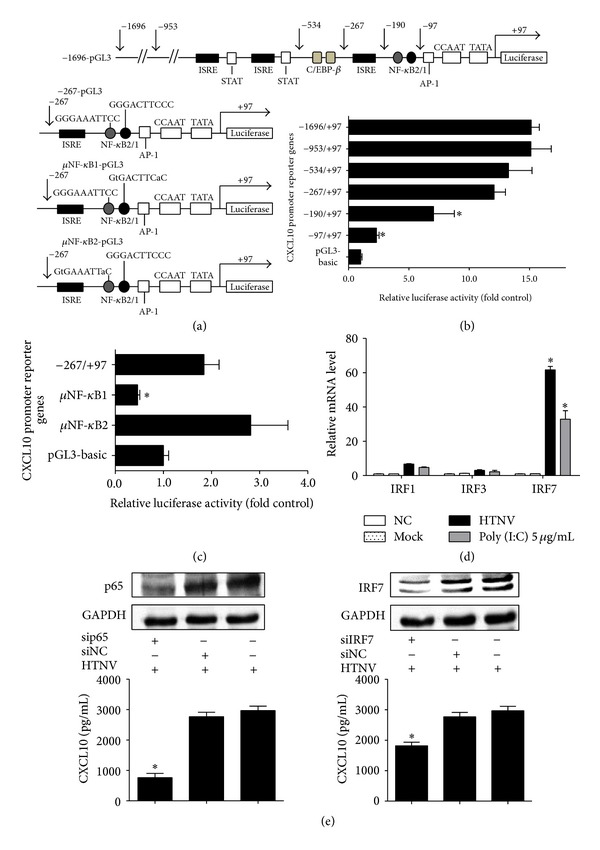
*NF-*κ*B1 and IRF7 upregulate CXCL10 expression.* (a) Schematic diagram of full-length, truncated, and mutated *CXCL10* promoter constructs. (b) Various truncated *CXCL10* promoter constructs were transfected into 293T cell lines, and the relative luciferase activities to the pGL3-basic vector were detected. The data showed that the NF-*κ*B and ISRE sites in −267/+97 construct were of importance in regulation of the transcription of *CXCL10* gene. (c) Influences of mutated NF-*κ*B sites on activity of the *CXCL10* promoter indicated that NF-*κ*B1 site may play a more important role. (d) Real-time PCR was used to prove that only IRF7 upregulated substantially at 48 h post-HTNV infection on HUVEC model. (e) When transfected into HUVECs for 24 h with siRNA (upper panel), specific to p65 or IRF7, respectively, and then infected the cells with HTNV (MOI = 1) for another 48 h, the expressions of CXCL10 reduced (lower panel). All the experiments were repeated in triplicate. mRNA data were generated from three independent experiments with three independent HUVECs donors. Data were means ± SE. *P < 0.05, *CXCL10* promoter versus pGL3-basic vector transfected cell; HTNV infected or poly (I:C) treated versus medium treated cells; and siRNA specific to p65 or IRF7, versus siNC treated cells.

Schematic diagram of full-length, truncated and mutated *CXCL10* promoter constructs is shown in Figure 4(a). The measurement of luciferase activity 48 h after transfection found that the deletions from nt −267 to nt −97, which contains two NF-*κ*B sites and one ISRE site, significantly reduced the CXCL10 transcription activity (Figure 4(b)). The respective mutation of the two NF-*κ*B sites showed that only mutation of NF-*κ*B1 binding site effectively reduced the activation of* CXCL10* promoter (Figure 4(c)). Real-time PCR analysis in HTNV-infected HUVEC model showed that only IRF7 increased markedly after HTNV infection and poly (I:C) transfection (Figure 4(d)). The knockdown of p65 or IRF7 in HTNV-infected HUVECs impaired the inductions of CXCL10 (Figure 4(e)).

### 3.5. p65 and IRF7 Translocate to Nucleus and Bind to CXCL10's Promoter after HTNV Infection

**Figure 5:**
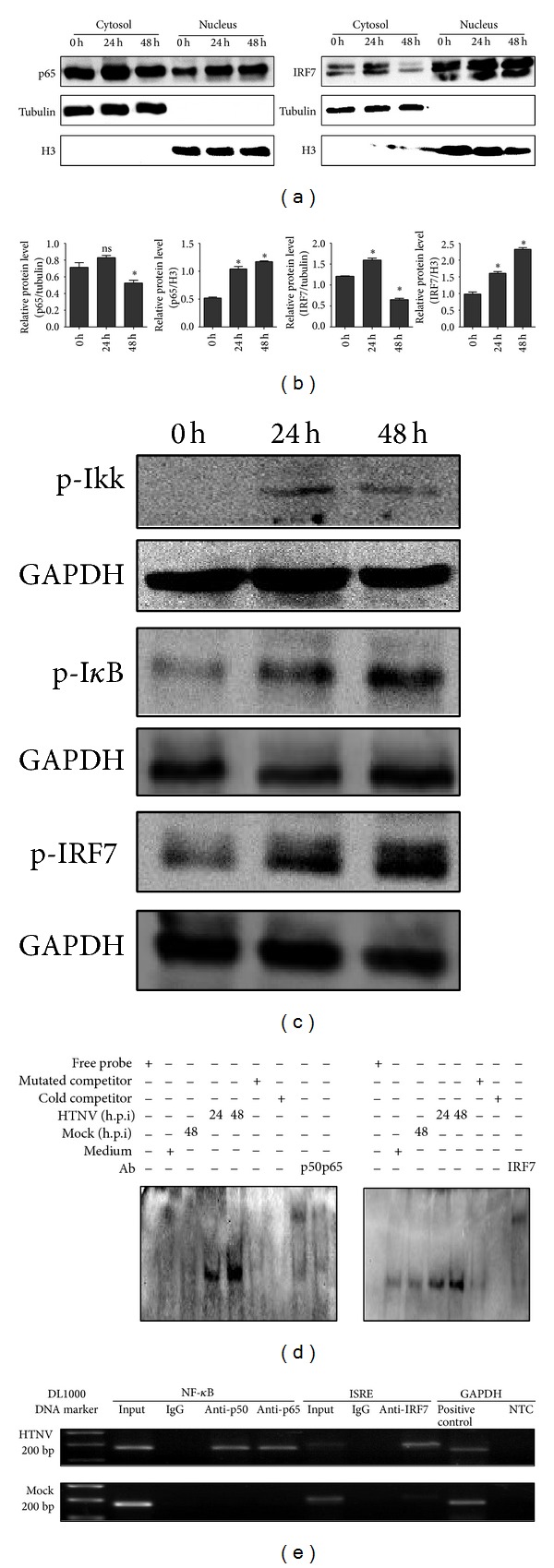
*p65 and IRF7 translocate to nucleus and bind to CXCL10's promoter after HTNV infection.* (a) WB results showed that HTNV infection induced the nuclear translocation of p65 and IRF7. Lysates were also immunoblotted using anti-*β*-tubulin or antihistone H3 (H3) as loading controls and to confirm the integrity of the cytosolic and nuclear lysates, respectively. (b) Semiquantitative analysis of p65 and IRF7 levels indicated in (a) was performed by Band-Scan software 5.0, and expression of *β*-tubulin or H3 was used as cytosolic or nuclear control, respectively. Data were means ± SE. *P < 0.05, 24 h or 48 h versus 0 h p.i with HTNV. (c) At 24 h and 48 h p.i, phosphorylation of Ikk, I*κ*B, and IRF7 can be detected by Western blot. (d) EMSA results showed direct bindings of p50 and p65 to the NF-*κ*B1 site (left) and IRF7 to the ISRE site (right) of *CXCL10* promoter after HTNV infection. Super shift bands were shown when the specific antibodies to p50, p65, and IRF7 were used, respectively. (e) ChIP assay also documented that p50, p65, and IRF7 bound directly to the *CXCL10* promoter. The upper panel showed that, at 48 h post-HTNV infection, both a 177-bp DNA fragment and a 200-bp DNA fragment containing the NF-*κ*B1 site and ISRE site in the *CXCL10* promoter, respectively, can be amplified successfully, while the mock virus cannot (lower panel). The input DNA and the positive (anti-polymerase antibody) and the negative control (normal mouse IgG) were used to verify the reliability of ChIP assay.

NF-*κ*B1 and IRF7 were translocated from cytosol to nucleus after HTNV infection (Figure 5(a)). The densitometric analysis of WB results showed the ratios of the protein levels of p65 and IRF7 relative to their respective loading control *β*-tubulin or histone H3 (Figure 5(b)). Western blot analysis found that the phosphorylation of Ikk, I*κ*B, and IRF7 could be detected at 24 h post infection (p.i) and 48 h p.i (Figure 5(c)). EMSA examination confirmed that NF-*κ*B1 and IRF7 were binding directly to the CXCL10 promoter region. The positive mobility shifts for both NF-*κ*B1 and IRF7 binding oligonucleotides were observed only when the oligonucleotides were incubated with the nuclear extracts of HTNV-infected (24 and 48 h p.i) but not in mock virus-infected or medium culture treated HUVECs (Figure 5(d)). The incubation of oligonucleotide-nuclear complexes respective with anti-p50, anti-p65, and anti-IRF7 antibodies prior to gel resolution and the super shift bands confirmed that the observed shifts resulted from p50, p65, and IRF7 bindings (Figure 5(d)).

ChIP examination proved that the transcriptional factors bound directly with *CXCL10* promoter region. Using DNA isolated from HTNV infected cell lysates as template, a 177-bp DNA fragment containing NF-*κ*B site and a 200-bp DNA fragment containing ISRE site were amplified in the presence of anti-p50, anti-p65, or anti-IRF7 antibodies, but they were not seen in the mock virus infection group or in the presence of control antibody (Figure 5(e)).

## 4. Discussion

The vital role of CXCL10 in the pathogenesis has been reported in many virus infectious diseases. Though Peng-Peng Ip and Fang Liao found that CXCL10 could resist Dengue virus (DENV) infection in mice because the susceptibility of CXCL10^−/−^ mice to DENV infection was enhanced [12], many other studies reported that the high values of CXCL10 levels could be used as a biomarker of disease progression and some in vivo studies showed that elevated levels of CXCL10 contributed to the tissue damages [7, 9, 24–28]. The contradictory findings of CXCL10's role in either protecting or promoting infection may depend on the host immune status and genetic background [29]. In our study, we have found that the serum CXCL10 level in HFRS patients is substantially higher than its corresponding level in healthy donors (Figure 1(a)) and the content of CXCL10 in the acute phase of HFRS is 3 times higher than that in the convalescent phase (Figure 1(b)). Importantly, the elevation of CXCL10 level is correlated significantly with the HFRS severity-indicating clinical parameters (Figures 1(c) and 1(d)). The higher CXCL10 level in the higher viral load group (>1500 copies/mL) is good proof that the production of CXCL10 is correlated with the replication of the HTNV (Figure 1(d)).

CXCL10-CXCR3 signaling axis recruit virus-specific T cells into inflammatory sites and promote viral clearance during virus infection [30]. Our analyses of the membrane expression level of CXCR3 on T cells, NK cells, and monocytes (data not shown) reveal that the level of CXCR3 increases on CD14^+^ subset in PBMCs isolated from HFRS patients (Figure 2). These results suggest that the increasing level of CXCL10 in the acute phase of HFRS may recruit CD14^+^ cells (monocytes) and the recruited CD14^+^ monocytes may produce excessive cytokines to be involved in the “cytokine storm.” Our presumption is consistent with the findings of Cheung et al. in their study on SARS-coronavirus [11]. These findings indicate that the elevated level of CXCL10 in HFRS patients may serve as a biomarker of HFRS development. Since there has not been a proper animal model for HFRS [5], we cannot yet testify the protective effect of antagonist on the CXCL10-CXCR3 signaling axis after HTNV infection in vivo.

JAK-STAT, TRAF2/TAK1, and PI3K/AKT signaling pathways are reported to be involved in the regulation of CXCL10 in such virus infectious diseases as HIV, HBV, and influenza A [7, 14, 15]. But, the mechanism for the induction of CXCL10 during HTNV infection is still unknown. In our study, the results of the transfection of poly (I:C) suggest that the CXCL10 could be induced by dsRNA, which may be the replicative intermediate generated during HTNV replication (Figure 3). As indicated in Figure 3(a), mock viruses, which lose their ability in replication, fail to activate the expression of CXCL10. The transfection of R848 also demonstrates that it is dsRNA, rather than ssRNA, that induces the CXCL10's production. dsRNA sensors (TLR3, RIG-I, and MDA-5) are activated after HTNV infection and therefore may contribute to the CXCL10's generation, while TLR7 and TLR8, as ssRNA sensors, are not activated (Figures 3(a)–3(c)). Sundstrom et al. reported that, during HTNV infection, IRF3 and IRF7, rather than IRF-1 or p65, were the main downstream transcription factors regulating the cytokines' production [31] but they did not explain the direct correlations between these transcription factors and CXCL10's production during HTNV infection. Our results of ChIP and EMSA demonstrate that IRF7 and NF-*κ*B1 are activated after HTNV infection and they bind directly to the CXCL10's promoter in HUVEC (Figure 5). The understanding of the regulations of cytokines/chemokines and their receptors during HTNV infection might provide a novel therapeutic target in HFRS. In our study, we have found that the contents of CXCL10 (10 kDa) in HFRS patients' serum drop to the normal level after the hemodialysis treatment (data not shown). Current treatment of HFRS is often limited to the administration of Ribavirin and primary supports [21, 32]. The patients suffering from a severe or critical HFRS have to be treated by hemodialysis [5] to remove the low molecular weight proteins, such as cytokines and chemokines [33]. But hemodialysis has some serious side effects, such as bleeding, hypotension, infection, and anemia. A combination of antiviral and anti-cytokines/chemokines therapies may be a better therapeutic approach to hantavirus infections [34].

Some reports contended that HTNV, as a member of negative ssRNA, produced undetectable amounts of dsRNA during the infection [35]. At present, the innate immunity activated by HTNV infection remains a subject of much debate with its focus on whether dsRNA like structures are produced during the replication process of HTNV infection. The disagreement may be due to the different cell lines used in respective studies. Some clinical and pathological findings have shown that pathogenic hantaviruses specifically target endothelial cells for infection [36–38] and accordingly in our study we used the HUVEC model. Further studies are still needed for hantaviral RNA structures that trigger innate immunity activity in vivo and their interactions with TLR3, RIG-I, and MDA-5 or with some other unknown RNA sensors.

## Supplementary Material

Table S1. Screen multiple cytokines in sera of HFRS patients using Luminex.
Table S2. Primer sequences for use in Real-time PCR.
Table S3. Primer sequences for the plasmids construction.
Table S4. Specific siRNA for use in RNA interference.

## References

[1] Schmaljohn CS, Dalrymple JM (1983). Analysis of Hantaan virus RNA: evidence for a new genus of Bunyaviridae. *Virology*.

[2] Lee HW, Lee RW, Johnson KM (1978). Isolation of the etiologic agent of Korean hemorrhagic fever. *Journal of Infectious Diseases*.

[3] Vaheri A, Strandin T, Hepojoki J (2013). Uncovering the mysteries of hantavirus infections. *Nature Reviews Microbiology*.

[4] Mackow ER, Gavrilovskaya IN (2009). Hantavirus regulation of endothelial cell functions. *Thrombosis and Haemostasis*.

[5] Schonrich G, Rang A, Lutteke N (2008). Hantavirus-induced immunity in rodent reservoirs and humans. *Nature Reviews Immunology*.

[6] Neville LF, Mathiak G, Bagasra O (1997). The immunobiology of interferon-gamma inducible protein 10 kD (IP-10): a novel, pleiotropic member of the C-X-C chemokine superfamily. *Cytokine and Growth Factor Reviews*.

[7] Zhou Y, Wang S, Ma J-W (2010). Hepatitis B virus protein X-induced expression of the CXC chemokine IP-10 is mediated through activation of NF-*κ*B and increases migration of leukocytes. *The Journal of Biological Chemistry*.

[8] Lagging M, Romero AI, Westin J (2006). IP-10 predicts viral response and therapeutic outcome in difficult-to-treat patients with HCV genotype 1 infection. *Hepatology*.

[9] Jiao Y, Zhang T, Wang R (2012). Plasma IP-10 is associated with rapid disease progression in early HIV-1 infection. *Viral Immunology*.

[10] Cameron CM, Cameron MJ, Bermejo-Martin JF (2008). Gene expression analysis of host innate immune responses during lethal H5N1 infection in ferrets. *Journal of Virology*.

[11] Cheung CY, Poon LLM, Ng IHY (2005). Cytokine responses in severe acute respiratory syndrome coronavirus-infected macrophages in vitro: possible relevance to pathogenesis. *Journal of Virology*.

[12] Ip P-P, Liao F (2010). Resistance to dengue virus infection in mice is potentiated by CXCL10 and is independent of CXCL10-mediated leukocyte recruitment. *Journal of Immunology*.

[13] Zaheer RS, Proud D (2010). Human rhinovirus-induced epithelial production of CXCL10 is dependent upon IFN regulatory factor-1. *American Journal of Respiratory Cell and Molecular Biology*.

[14] Williams R, Yao H, Dhillon NK, Buch SJ (2009). HIV-1 Tat co-operates with IFN-*γ* and TNF-*α* to increase CXCL10 in human astrocytes. *PLoS ONE*.

[15] Lu X, Masic A, Liu Q, Zhou Y (2011). Regulation of influenza A virus induced CXCL-10 gene expression requires PI3K/Akt pathway and IRF3 transcription factor. *Molecular Immunology*.

[16] Ji Y, Liu J, Wang Z, Li Z (2011). PPAR*γ* agonist rosiglitazone ameliorates LPS-induced inflammation in vascular smooth muscle cells via the TLR4/TRIF/IRF3/IP-10 signaling pathway. *Cytokine*.

[17] Geimonen E, Neff S, Raymond T, Kocer SS, Gavrilovskaya IN, Mackow ER (2002). Pathogenic and nonpathogenic hantaviruses differentially regulate endothelial cell responses. *Proceedings of the National Academy of Sciences of the United States of America*.

[18] Yi J, Xu Z, Zhuang R (2013). Hantaan virus RNA load in patients having hemorrhagic fever with renal syndrome: correlation with disease severity. *The Journal of Infectious Diseases*.

[19] Wang M, Wang J, Zhu Y (2009). Cellular immune response to hantaan virus nucleocapsid protein in the acute phase of hemorrhagic fever with renal syndrome: Correlation with disease severity. *Journal of Infectious Diseases*.

[20] Liu B, Ma Y, Yi J (2013). Elevated plasma soluble sema4D/CD100 levels are associated with disease severity in patients of hemorrhagic fever with renal syndrome. *PLoS ONE*.

[21] Jonsson CB, Figueiredo LTM, Vapalahti O (2010). A global perspective on hantavirus ecology, epidemiology, and disease. *Clinical Microbiology Reviews*.

[22] Md Sheikh A, Ochi H, Manabe A, Masuda J (2005). Lysophosphatidylcholine posttranscriptionally inhibits interferon-*γ*- induced IP-10, Mig and I-Tac expression in endothelial cells. *Cardiovascular Research*.

[23] Kim Y, Fischer SM (1998). Transcriptional regulation of cyclooxygenase-2 in mouse skin carcinoma cells: Regulatory role of CCAAT/enhancer-binding proteins in the differential expression of cyclooxygenase-2 in normal and neoplastic tissues. *The Journal of Biological Chemistry*.

[24] Roe B, Coughlan S, Hassan J (2007). Elevated serum levels of interferon-*γ*-inducible protein-10 in patients coinfected with hepatitis C virus and HIV. *Journal of Infectious Diseases*.

[25] Kolb SA, Sporer B, Lahrtz F, Koedel U, Pfister H-W, Fontana A (1999). Identification of a T cell chemotactic factor in the cerebrospinal fluid of HIV-1-infected individuals as interferon-*γ* inducible protein 10. *Journal of Neuroimmunology*.

[26] Butera D, Marukian S, Iwamaye AE (2005). Plasma chemokine levels correlate with the outcome of antiviral therapy in patients with hepatitis C. *Blood*.

[27] Spurrell JCL, Wiehler S, Zaheer RS, Sanders SP, Proud D (2005). Human airway epithelial cells produce IP-10 (CXCL10) in vitro and in vivo upon rhinovirus infection. *American Journal of Physiology*.

[28] Kim YH, Kim J-E, Hyun MC (2011). Cytokine response in pediatric patients with pandemic influenza H1N1 2009 virus infection and pneumonia: comparison with pediatric pneumonia without H1N1 2009 infection. *Pediatric Pulmonology*.

[29] Deng G, Zhou G, Zhang R (2008). Regulatory polymorphisms in the promoter of CXCL10 gene and disease progression in male hepatitis B virus carriers. *Gastroenterology*.

[30] Lindell DM, Lane TE, Lukacs NW (2008). CXCL10/CXCR3-mediated responses promote immunity to respiratory syncytial virus infection by augmenting dendritic cell and CD8+ T cell efficacy. *European Journal of Immunology*.

[31] Sundstrom JB, McMullan LK, Spiropoulou CF (2001). Hantavirus infection induces the expression of RANTES and IP-10 without causing increased permeability in human lung microvascular endothelial cells. *Journal of Virology*.

[32] Huggins JW, Hsiang CM, Cosgriff TM (1991). Prospective, double-blind, concurrent, placebo-controlled clinical trial of intravenous ribavirin therapy of hemorrhagic fever with renal syndrome. *Journal of Infectious Diseases*.

[33] Silvester W (1997). Mediator removal with CRRT: complement and cytokines. *American Journal of Kidney Diseases*.

[34] Maes P, Clement J, Gavrilovskaya I, van Ranst M (2004). Hantaviruses: immunology, treatment, and prevention. *Viral Immunology*.

[35] Wang H, Vaheri A, Weberspi-Sup F, Plyusnin A (2011). Old world hantaviruses do not produce detectable amounts of dsRNA in infected cells and the 59 termini of their genomic RNAs are monophosphorylated. *Journal of General Virology*.

[36] Nolte KB, Feddersen RM, Foucar K (1995). Hantavirus pulmonary syndrome in the United States: a pathological description of a disease caused by a new agent. *Human Pathology*.

[37] Pensiero MN, Sharefkin JB, Dieffenbach CW, Hay J (1992). Hantaan virus infection of human endothelial cells. *Journal of Virology*.

[38] Lahdevirta J (1982). Clinical features of HFRS in Scandinavia as compared with East Asia. *Scandinavian Journal of Infectious Diseases*.

