# Supplementary material for: Hantaan Virus Infection Induces CXCL10 Expression through TLR3, RIG-I, and MDA-5 Pathways Correlated with the Disease Severity
Source: Mediators Inflamm. 2014 Feb 23;2014:697837. doi: 10.1155/2014/697837 (PMC3950924; doi:10.1155/2014/697837)
Supplement: Supplementary file 1 — Table S1. Screen multiple cytokines in sera of HFRS patients using Luminex. Table S2. Primer sequences for use in Real-time PCR. Table S3. Primer sequences for the plasmids construction. Table S4. Specific siRNA for use in RNA interference. [file 697837.f1.doc]

**Supporting Information**

**Table S1.**

|  | NC (n=20) | | HFRS (n=90) | |  |  |
| --- | --- | --- | --- | --- | --- | --- |
|  | medium (pg/ml) | SD | Medium  (pg/ml) | SD | p | Folds  (HFRS vs. NC) |
| IFN-γ | 9.77 | 0.21 | 245.63 | 849.69 | 0.22 | 25.14 |
| IL-13 | 9.65 | 0.20 | 17.92 | 37.93 | 0.36 | 1.86 |
| IL-17 | 156.25 | 10.76 | 1435.72 | 2273.92 | 0.01 | 9.19 |
| IL-17A | 16.18 | 4.52 | 22.14 | 72.71 | 0.72 | 1.37 |
| IL-1β | 47.39 | 24.03 | 74.21 | 385.11 | 0.76 | 1.57 |
| IL-1RA | 9.69 | 0.23 | 182.62 | 482.82 | 0.11 | 18.85 |
| IL-2 | 89.16 | 4.52 | 141.65 | 355.75 | 0.54 | 1.59 |
| IL-5 | 9.70 | 4.01 | 19.77 | 86.24 | 0.60 | 2.04 |
| IL-7 | 14.65 | 0.46 | 323.14 | 871.56 | 0.12 | 22.06 |
| CXCL10 | 279.97 | 195.22 | 6991.43 | 6978.39 | <0.001 | 24.97 |
| MIP-1β | 278.96 | 121.02 | 263.69 | 252.35 | 0.79 | 0.95 |
| VEGF | 23.1 | 6.83 | 162.55 | 290.38 | 0.04 | 7.04 |

**Table S2.**

| Gene | Forward primers (5’-3’) | Backward primers (5’-3’) |
| --- | --- | --- |
| CXCL10 | ACCTCCAGTCTCAGCACCAT | TCAGAAAGATAAGGCAGCAA |
| TLR3 | ACATACTCAACCTAACCA | AAAGGAGTTCCTAGTCAG |
| TLR7 | TCCTTGGGGCTAGATGGTTTC | TCCACGATCACATGGTTCTTTG |
| TLR8 | TGTGATGGTGGTGCTTCAAT | ATGCCCCAGAGGCTATTTCT |
| RIG-I | CTGGACCCTACCTACATCCTG | GGCATCCAAAAAGCCACGG |
| MDA-5 | GCCCGCTACATGAACCCTG | CAGCAATCCGGTTTCTGTCTT |
| IRF1 | GACCAGAGCAGGAACAAGGG | CTCGGCTGGACTTCGACTTT |
| IRF3 | CCCCAAGGACAAGGAAGGAG | CAGAACCAGAGGGCATAGCG |
| IRF7 | TACCATCTACCTGGGCTTCG | AGGGTTCCAGCTTCACCA |
| GAPDH | GACCTGACCTGCCGTCTA | AGGAGTGGGTGTCGCTGT |

**Table S3**.

| plasmid | Forward primers (5’-3’) | Backward primers (5’-3’) |
| --- | --- | --- |
| -1696/+97-pGL3 basic | GGGGTACCTAAGACTTTCCCTCATCAGAA | AAACTCGAGGGCAGCAAATCAGAATGGCA |
| -953/+97-pGL3 basic | TTTGCTAGCCAGTTATCACTGTTACTAGC | AAACTCGAGGGCAGCAAATCAGAATGGCA |
| -534/+97-pGL3 basic | TTTGCTAGCACTTGCCAGTTCCAGATCTT | AAACTCGAGGGCAGCAAATCAGAATGGCA |
| -267/+97-pGL3 basic | TTTGCTAGCCTATATGCAATGAAGTTCTT | AAACTCGAGGGCAGCAAATCAGAATGGCA |
| -190/+97-pGL3 basic | TTTGCTAGCAAAAGAGGAGCAGAGGGAAA | AAACTCGAGGGCAGCAAATCAGAATGGCA |
| -97/+97-pGL3 basic | TTTGCTAGCGGTTTTGCTAAGTCAACTGT | AAACTCGAGGGCAGCAAATCAGAATGGCA |

**Table S4.**

| Target gene | Sense (5’-3’) | Antisense (5’-3’) |
| --- | --- | --- |
| siTLR3 | CGAAUUUGACUGAACUCCATT | UGGAGUUCAGUCAAAUUCGTG |
| siRIG-I | GGCGUUCUCUAGAUCCUUUTT | AAAGGAUCUAGAGAACGCCTG |
| siMDA-5 | GGUGUAAGAGAGCUACUAATT | UUAGUAGCUCUCUUACACCTG |
| sip65 | GCCUUAAUAGUAGGGUAAGTT | AACUUACCCUACUAUUAAGTG |
| siIRF7 | GCUGGACGUGACCAUCAUGUATT | UACAUGAUGGUCACGUCCAGCTG |
| siNC | UUCUCCGAACGUGUCACGUTT | ACGUGACACGUUCGGAGAATT |
